# Interleukin-6 Levels in Women with Polycystic Ovary Syndrome: A Systematic Review and Meta-Analysis

**DOI:** 10.1371/journal.pone.0148531

**Published:** 2016-02-05

**Authors:** Zheng Peng, Yifan Sun, Xiaolan Lv, Hongyu Zhang, Chunming Liu, Shengming Dai

**Affiliations:** 1 Department of Clinical Laboratory, the Fourth Affiliated Hospital of Guangxi Medical University, Liuzhou, Guangxi, China; 2 Department of Clinical Laboratory, Liuzhou Hospital of Traditional Chinese Medicine, Liuzhou, Guangxi, China; 3 Department of Clinical Laboratory, Liuzhou Maternal and Child Health Care Hospital, Liuzhou, Guangxi, China; Medical University Innsbruck, AUSTRIA

## Abstract

**Background:**

The change of serum interleukin-6(IL-6) levels in women with polycystic ovary syndrome (PCOS), as well as the relations between IL-6 levels and body mass index (BMI), insulin resistance(IR) and androgen status of PCOS patients, are not fully understood.

**Methods:**

A literature search was performed in October 2015 using PubMed, Embase and the Cochrane Library databases to identify studies. Random-effects model was used to estimate the standardized mean differences (SMDs) with 95% confidence intervals (CIs).

**Results:**

Twenty articles with 25 case-control studies included 1618 women (922 PCOS patients and 696 controls) were included in this study. IL-6 levels in controls were significantly lower than that of PCOS patients (SMD = 0.78, 95%CI = 0.41–1.16, P<0.001), with significant heterogeneity across studies (*I*^*2*^ = 91% and P<0.001). Meta-regression analysis model indicated IR status was the main source of heterogeneity (*P* = 0.005). Results from group analysis suggested that high IL-6 levels in PCOS were significantly associated with Homeostasis Model Assessment of Insulin Resistance (HOMA2-IR) ratio and total testosterone ratio (T ratio), and was found in both lean and obese women with PCOS. Cumulative meta-analysis results indicated the total effect size (SMD) had tend to be stable since 2012(0.79 to 0.92).

**Conclusions:**

A high IL-6 level is not an intrinsic characteristic of PCOS, but may be a useful monitoring biomarker for the treatment of PCOS.

## Introduction

Polycystic ovary syndrome (PCOS) is a common endocrine disorder, which affects about one in 15 women worldwide [1]. According to the Rotterdam criteria [2] and National Institute of Health (NIH) criteria [3], hyperandrogenism, chronic anovulation, and polycystic ovaries on ultrasonography are the three main clinical features of PCOS patients. PCOS is a complex heterogeneous disease, and obesity, insulin resistance (IR), and metabolic syndrome are common in women with PCOS [1]. Therefore, it is not surprising that individuals with PCOS have an increased lifetime risk of type 2 diabetes (T2DM) and cardiovascular diseases.

Low-grade chronic inflammation in women with PCOS is involved in the pathogenesis of T2DM and cardiovascular disease [1, 4]. Interleukin-6 (IL-6), a major proinflammatory cytokine in chronic inflammation, has been shown to be closely associated with IR and cardiovascular abnormalities [5, 6]. Early research *in vivo* showed that infusion of human recombinant IL-6 could induce gluconeogenesis, subsequent hyperglycemia, and compensatory hyperinsulinemia [7]. Obesity, a major risk factor for T2DM, was reported to be associated with elevated IL-6 levels [8, 9]. In contrast, IL-6 levels decreased in PCOS patients after they reduced their level of IR and body mass [10]. IL-6 might play a key role in the development of cardiovascular disease through metabolic, endothelial, and coagulant [5]. Elevated levels of IL-6 were reported to be associated with an increased risk of future myocardial infarction and atherothrombosis [11, 12].

The data presented above illustrate that IL-6 is a key mediator, which is linked to T2DM and cardiovascular diseases in women with PCOS. Therefore, IL-6 may be a useful biomarker for the diagnosis of PCOS and the treatment of T2DM and cardiovascular diseases in women with PCOS. However, the findings of recent studies of changes in IL-6 levels in PCOS patients are inconsistent. Although some studies reported significant elevations in IL-6 levels in women with PCOS compared with controls [9, 13–20], these were not confirmed in similar studies [21–27], with some studies even reporting decreased IL-6 levels [28, 29].

Two meta-analyses of IL-6 levels in PCOS were reported in 2011 [30, 31], and further studies on this topic were conducted after 2011 [18, 20, 27, 32]. Moreover, these studies did not report the relations between IL-6 levels and the baseline characteristics of those with PCOS, such as their body mass index (BMI), level of IR, and androgen status. Thus, the purpose of the present study was to conduct a systematic review of the literature on serum IL-6 levels in women with PCOS and to investigate the impact of the characteristics of PCOS on IL-6 levels using a meta-analysis.

## Material and Methods

The meta-analysis, including the search strategy, selection criteria, data extraction, and data analysis(S1 Table), was implemented in accordance with the PRISMA statement (Preferred Reporting Items for Systematic Reviews and Meta-Analyses) [33].

### Search Strategy

A literature search to identify relevant studies was performed in October 2015 using PubMed, Embase, and the Cochrane Library database. The following search terms were used: “interleukin-6,” “IL-6,” “IL 6,” “polycystic ovary syndrome,” and “PCOS”. The searches were restricted to studies of only human subjects, and no language restrictions were applied (S1 File). In addition, the reference lists of the identified studies and those of the meta-analyses were searched manually to identify relevant articles [30, 31]. Two reviewers (YS and ZP) independently searched the electronic databases, and they screened the titles, abstracts, and full-text articles after excluding duplicated records. Any discrepancy in the screening process was resolved by consultation of the group.

#### Study selection

Studies that reported the IL-6 levels of PCOS patients were selected. The inclusion criteria were as follows:

1. The study included a PCOS group and BMI-matched control group.
2. The diagnosis of PCOS was made according to Rotterdam criteria [2], or NIH criteria [3].

Studies that included subjects with diseases other than PCOS and that measured IL-6 levels in tissue or IL-6 mRNA levels were excluded. Studies lacking data on IL-6 means and their standard deviations (SDs) (and this information could not be obtained from the authors) and studies with a sample size of less than 10 were excluded. Letters, case reports, editorials, and conference abstracts were also excluded.

### Quality Score Assessment

To assess the quality of each study, the Newcastle-Ottawa Scale [34] was used, with some modification. The predefined criteria of the scale are shown in S2 Table. According to the quality score assessment, the distribution of the scores was between 0 and 7. In a case where the score of a study was ≤4, it was classified as a “low-quality” study. If a study scored ≥5, it was considered a “high-quality” study.

### Data Extraction

Two of the authors (ZP and YS) independently extracted the data from the included studies. The general characteristics of the study were extracted using a standardized data extraction form: name of the first author, year of publication, country, ethnicity of the study population, number of the PCOS group and control group. Further, the following data of the PCOS and control groups were extracted from each study: diagnostic criteria, BMI, age, IL-6 levels and measurement method, insulin sensitivity status, and testosterone levels. If the two investigators could not reach an agreement, the dispute was resolved by a third reviewer (SD).

### Statistical Analysis

IL-6 levels were extracted as the mean difference ±SD in each study. In this meta-analysis, to better understand the nature of the difference in IL-6 levels in each study and remove any heterogeneity caused by different methods used to measure IL-6 levels, the IL-6 levels in the PCOS group were normalized using the control group as a reference. The normalization procedure was performed by dividing the mean level and associated SD in the PCOS group by the mean levels in the control reference group. The assessment of IR was based on reported mean glucose and insulin values, and Homeostasis Model Assessment of Insulin Resistance (HOMA2-IR) values were used to measure the degree of IR according to the formula on the website: www.dtu.ox.ac.uk (the Oxford Diabetes Trials Unit calculator). The relative between-group difference in IR in a study was expressed as the ratio of the mean HOMA2-IR value (HOMA2-IR ratio) in the PCOS group to that of the controls. Similarly, the relative difference in testosterone levels, which was used to measure the androgen status, was expressed by the ratio of mean total testosterone (T ratio) in PCOS women to controls.

If the mean level difference were large across studies, or different units were used, standardized mean difference (SMD) is more suitable than weighted mean difference (WMD) to estimate the effect size because WMD can only be used when all studies are made on the same scale. In this study, the serum IL-6 were measured in different assays, techniques and units across studies, and the differences in the mean levels of IL-6 were too large, therefore, SMD not WMD in plasma IL-6 was used to estimate the effect size. Heterogeneity was assessed using a chi-squared Q test and I-squared statistics. If *P*_*Q*_<0.1 or *I*^*2*^>50%, the heterogeneity was considered significant, and a random-effects model (the DerSimonian and Laird method) was used. Otherwise, a fixed-effects model was used.

When the results revealed statistically significant heterogeneity, possible explanations were investigated by a subgroup analysis according to the following: BMI, HOMA2-IR ratio, T ratio, and study quality using predefined criteria. The BMI was categorized into two groups: a lean group (BMI of <25 kg/m^2^) and an obese group (BMI of ≥25 kg/m^2^). The HOMA2-IR ratio and T ratio were categorized according to quartile intervals. A meta-regression analysis was performed to investigate the potential impact of the predefined study characteristics on the SMD of IL-6 levels. The SMD was used as the dependent variable, and the BMI, study quality, HOMA2-IR ratio, T ratio, and sample size were used as explanatory covariates. A multivariable analysis was further performed if the variables were significant at the 0.1 level.

A sensitivity analysis, with the studies omitted one by one, was performed to examine the influence of individual studies. A cumulative sequential meta-analysis of the studies was performed according to their year of publication. RevMan 5.2.7 (Cochrane Collaboration) and STATA software (version 12.0; Stata Corporation, College Station, TX) were used in this meta-analysis.

## Results

### Literature Selection

**Fig 1:**
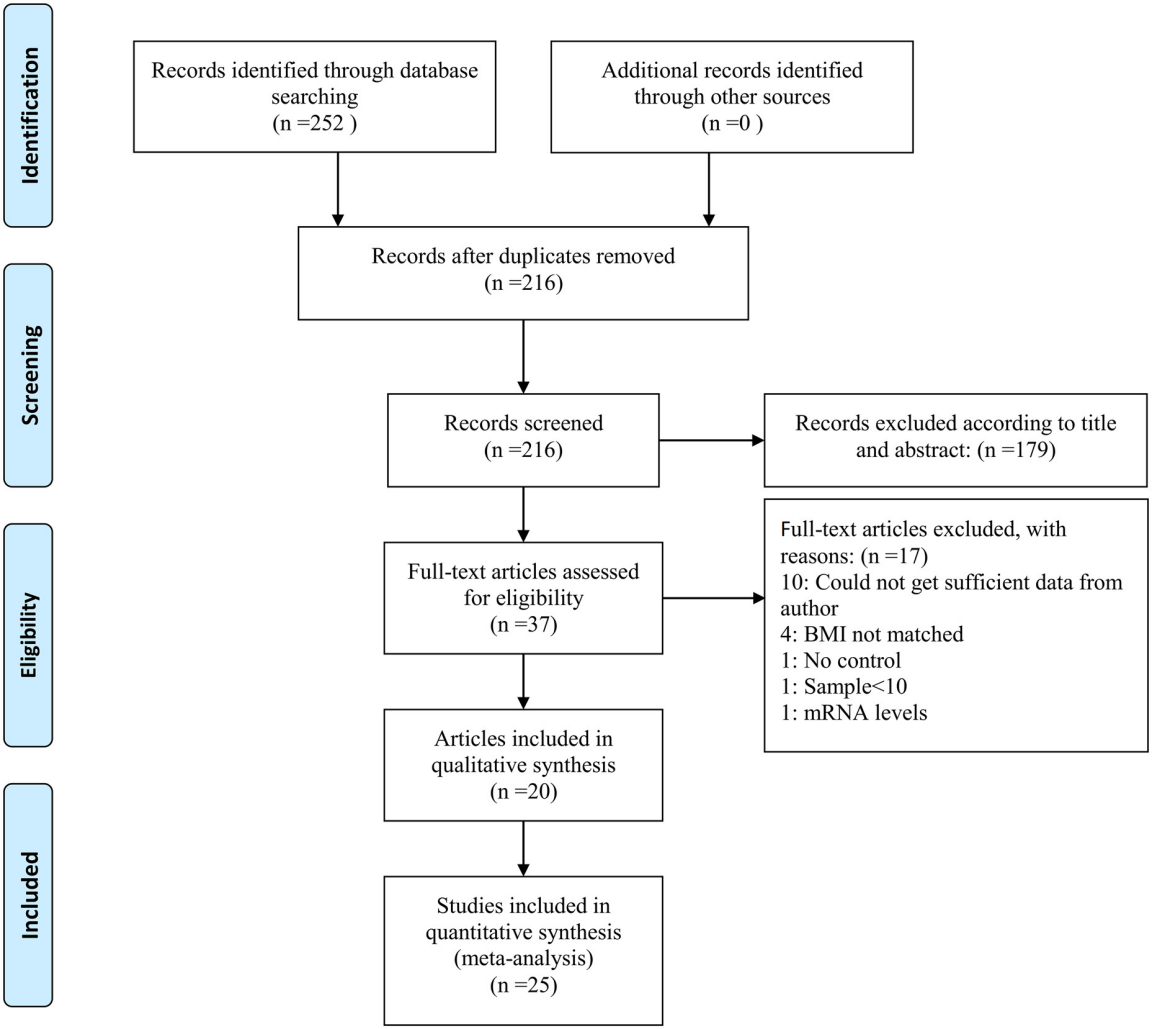
Flow diagram of included studies for this meta-analysis.

Two hundred fifty-two potentially relevant studies were identified according to the search strategy. After removing duplicated records and reviewing the titles and abstracts, 37 articles were included in the study. Of these, 17 articles that did not fulfill the selection criteria were excluded (S1 File). The BMI data in five articles were separated into two groups: a low BMI group (<25 kg/m^2^) and a high BMI group (≥25 kg/m^2^) [15, 16, 20, 32, 35], therefore, these five articles were separated as 10 studies. Finally, 20 articles (*n* = 25 studies) were included in this meta-analysis [9, 13–29, 32, 35]. Fig 1 shows the flowchart summarizing results.

### Systematic Review

Overall, the baseline characteristics, inclusion criteria, matched factors, and IL-6 levels were generally clearly stated in the included studies (Table 1 and S3 Table). The 25 case-control studies included 1618 participants (922 PCOS patients and 696 controls). Twenty of the reports were conducted in Caucasian populations [9, 13–24, 26–29, 32, 35], and only one study was conducted in an Asian population [25]. For the diagnosis of PCOS, seven of the studies used NIH criteria, and the other 18 used Rotterdam criteria. The major matched factors for the PCOS and control group were BMI, age, waist circumference, and smoking. Fourteen of the studies included PCOS patients with a BMI of ≥25 kg/m^2^, and 11 of the studies included PCOS patients with a BMI of <25 kg/m^2^. All the studies used an enzyme-linked immunosorbent assay to measure the IL-6 levels. However, the mean IL-6 levels were significantly different across studies (S3 Table). Twelve of the studies found higher IL-6 levels in the PCOS group compared with the controls, and 11 found no statistically significant difference in these levels. Only two of the studies reported decreased IL-6 levels in the PCOS patients. In this meta-analysis, according to the quality score assessment, five of the reports (*n* = 8 studies) [9, 14, 22, 32, 35] were classified as low quality, and the other 15 (*n* = 17 studies) [13, 15–21, 23–29] were considered high quality (Table 1).

**Table 1:** Characteristics of studies included in the meta-analysis.

Study	Year	Country	Diagnostic criteria	N_PCOS_	N_Control_	Matched factors	BMI (kg/m^2^)	IL6 Level	Quality Score
Escobar-Morreale	2003	Spain	NIH	35	28	BMI, Obesity, Smoking	≥25	NS	5
Tarkun	2006	Turkey	Rotterdam	32	25	BMI, Age, Waist circumference	<25	↑	5
Vgontzas	2006	USA	NIH	42	17	BMI, Age	≥25	↑	4
Moran	2007	Australia	Rotterdam	15	17	BMI, Smoking	≥25	NS	4
Olszanecka	2007	Poland	NIH	39	34	BMI, Age	≥25	↓	6
Glintborg	2008	Denmark	NIH	30	14	BMI, Age	≥25	↑	4
Jakubowska	2008	Poland	Rotterdam	29	29	BMI	≥25	NS	5
Gen	2009	Turkey	Rotterdam	21	15	Age, BMI, Waist circumference	<25	NS	5
Li	2009	China	Rotterdam	24	26	Age, BMI, Waist circumference	<25	NS	6
Samy	2009	Egypt	Rotterdam	52	40	BMI, Age	≥25	↑	5
Samy	2009	Egypt	Rotterdam	56	35	BMI, Age	<25	NS	5
Soares	2009	Brazil	Rotterdam	40	50	BMI, Age	<25	↓	6
Tsilchorozidou	2009	UK	NIH	30	18	BMI, Age	<25	NS	4
Tsilchorozidou	2009	Spain	NIH	29	18	BMI, Age	≥25	↑	4
Luque-Ramirez	2010	Poland	NIH	34	18	Age, BMI, Smoking, Waist circumference	≥25	NS	6
Nikolajuk	2010	Poland	Rotterdam	35	18	BMI, Age	<25	NS	5
Nikolajuk	2010	UK	Rotterdam	43	16	BMI, Age	≥25	↑	5
Victor	2011	Spain	Rotterdam	39	43	BMI, Age, Waist circumference	<25	↑	5
Ozcaka	2012	Turkey	Rotterdam	31	12	BMI	<25	↑	3
Gozdemir	2013	Turkey	Rotterdam	20	20	BMI, Age	<25	NS	4
Gozdemir	2013	Turkey	Rotterdam	20	20	BMI, Age	≥25	↑	4
Heutling	2013	Germany	Rotterdam	83	39	BMI, Age	≥25	NS	5
Phelan	2013	Ireland	Rotterdam	103	104	BMI	≥25	↑	5
Kucuk	2014	Turkey	Rotterdam	20	32	BMI	<25	↑	6
Kucuk	2014	Turkey	Rotterdam	20	8	BMI	≥25	↑	5

NIH, National Institutes of Health; BMI, body mass index; IL-6, interleukin-6; NS, no significant difference; ↑ = increased; ↓ = decreased; Low quality ≤4; High quality ≥5

### Meta-Analysis

#### Pooled analysis

**Fig 2:**
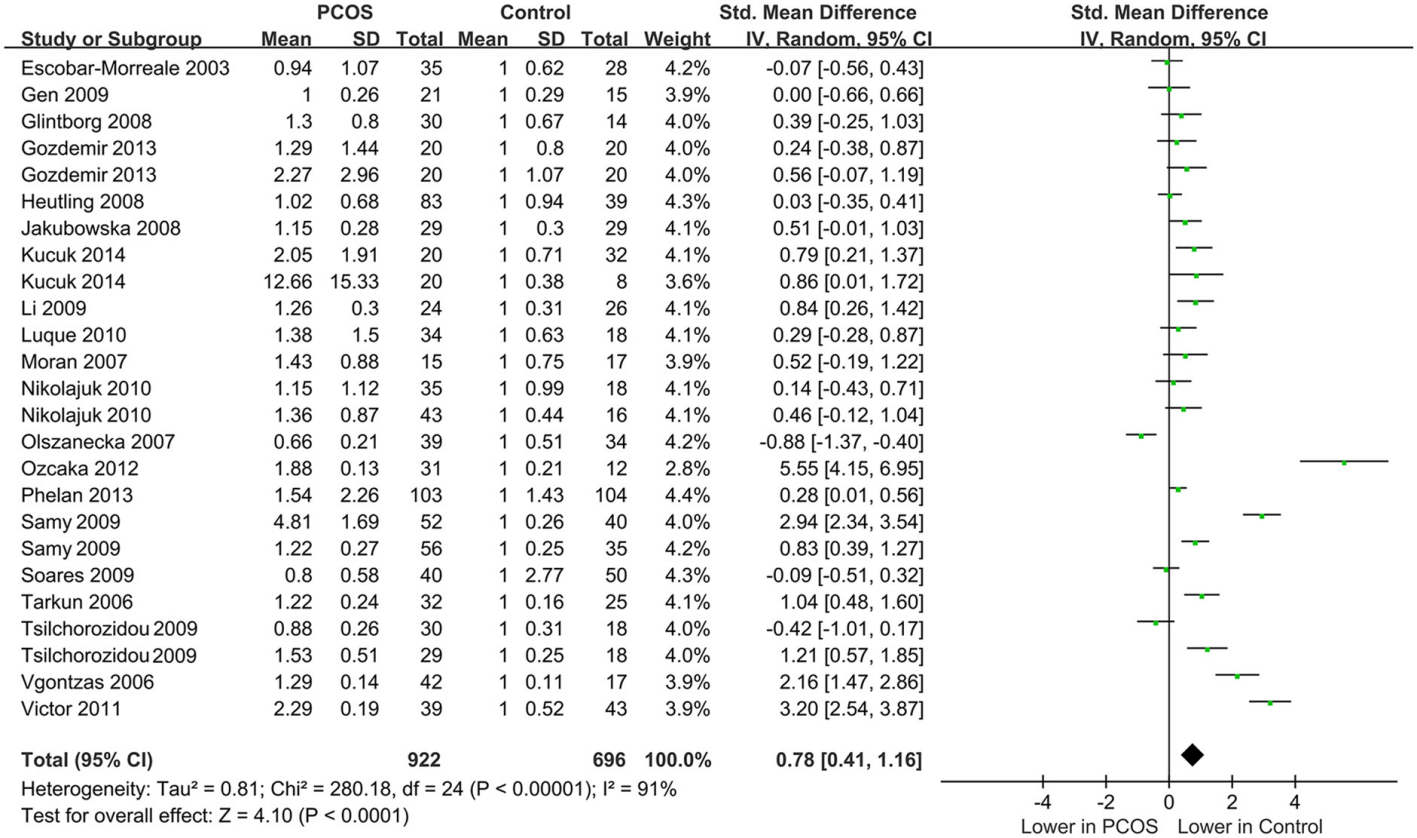
The pooled quantitative synthesis for IL-6 levels in PCOS patients compared with controls.

Of the 25 included studies, when all the data were pooled in the meta-analysis, the IL-6 levels of the controls were significantly lower than those of the PCOS patients (random-effects, SMD = 0.78, 95% CI = 0.41–1.16, *P*<0.001; Fig 2). However, significant heterogeneity was found across the included studies (*I*^*2*^ = 91% and *P*<0.001).

#### Subgroup analysis

**Fig 3:**
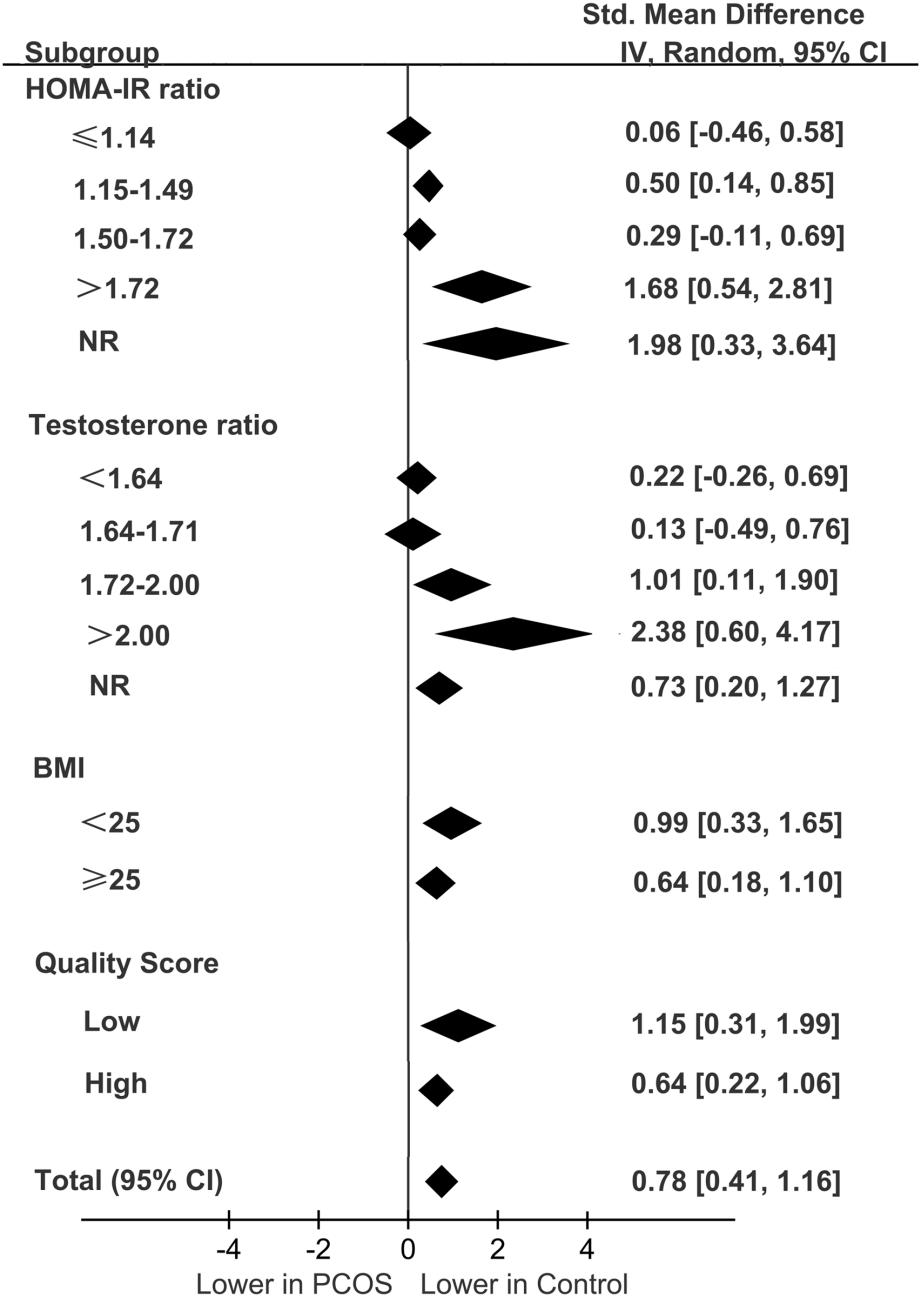
Summary of categorical meta-analysis.

A subgroup analysis was carried out according to the different categories of BMI and the HOMA2-IR ratio, T ratio, and study quality. The quartile intervals for the HOMA2-IR ratio were ≤1.14, 1.15–1.49, 1.50–1.72, and >1.72. The quartile intervals for T the ratio were <1.64, 1.64–1.71, 1.72–2.00, and >2.00. Studies with data of HOMA2-IR and total testosterone not reported or not available were categorized as the NR group. The results of the subgroup analysis are shown in Fig 3.

In the subgroup analysis, significant differences in the IL-6 levels of the PCOS patients versus the controls were observed in the quartiles with HOMA2-IR ratios of .15–1.49 and >1.72 (SMD = 0.50, 95% CI = 0.14–0.85, *P* = 0.006 and SMD = 1.68, 95% CI = 0.54–2.81, *P* = 0.004, respectively). However, there was no significant difference in the IL-6 levels of the quartiles with HOMA2-IR ratios of ≤1.14 and 1.50–1.72 categories (S1 Fig).

In the subgroup analysis, there was a significant difference in the IL-6 levels observed in the quartiles with high T ratios (T ratio = 1.72–2.00: SMD = 1.01, 95% CI = 0.11–1.90, *P* = 0.03 and T ratio >2.00: SMD = 2.38, 95% CI = 0.604.17, *P* = 0.009) but not in the quartiles with a low T ratio of <1.72 (data not shown, S2 Fig).

With regard to the subgroup analysis stratified by BMI and study quality, significantly lower IL-6 levels were associated with BMIs of <25 kg/m^2^ and ≥25 kg/m^2^ in the controls (SMD = 0.99, 95% CI = 0.33–1.65, *P* = 0.003 and SMD = 0.64, 95%CI = 0.18–1.10, *P* = 0.006, respectively; S3 Fig) but not in the PCOS patients with low and high BMIs using a random-effects model (*I*^*2*^ = 93%, *P*<0.001 and *I*^*2*^ = 90%, *P*<0.001, respectively). There were also significant differences in the reported IL-6 levels of low-quality studies (SMD = 1.15, 95% CI = 0.31–1.99, *P* = 0.007 and *I*^*2*^ = 92%, *P*<0.001 for heterogeneity) and high-quality studies (SMD = 0.64, 95% CI = 0.22–1.06, *P* = 0.003 and *I*^*2*^ = 92%, *P*<0.001 for heterogeneity; S4 Fig).

#### Meta-regression analysis

A univariate meta-regression analysis indicated that the regression coefficients of the HOMA2-IR ratio and T ratio were significant at the level of 0.1 (*P* = 0.002 and *P* = 0.061, respectively). Therefore, these two covariates were entered in a multivariate meta-regression analysis. The regression coefficients of the HOMA2-IR ratio were still significant (*P* = 0.005) but not those of the T ratio (*P* = 0.835), indicating that the difference in IR between the PCOS and control group contributed significantly to the heterogeneity across the studies. According to the multivariate model, IR could explain 43.4% of the heterogeneity. The results of the meta-regression analysis are shown in Table 2.

**Table 2:** Univariate meta-regression analysis for the potential variables between studies.

Covariates	No. Studies	Coefficient	Standard error	*t*	*P*	95%CI
**Univariate meta-regression analysis**						
Score	25	0.548	0.564	0.97	0.340	[-0.617, 1.715]
BMI	25	-0.206	0.532	-0.39	0.703	[-1.306,0.895]
HOMA-IR ratio	21	1.193	0.332	3.59	0.002	[0.498,1.889]
T ratio	19	1.620	0.806	2.01	0.061	[-0.082,3.322]
Sample size	25	-0.002	0.007	-0.26	0.796	[-0.017, 0.013]
**Multivariate meta-regression analysis**						
HOMA-IR ratio	18	1.394	0.423	3.30	0.005	[0.492, 2.296]
T ratio	18	-0.125	0.589	-0.21	0.835	[-1.381, 1.132]
cons	18	-1.370	1.002	-1.37	0.192	[-3.506, 0.765]
REML estimate of between-study variance: tau2 = 0.5548						
Proportion of between-study variance explained: *I*^*2*^ = 86.42%, Adjust R^2^ = 43.40%						

BMI, body mass index; HOMA-IR, Homeostasis Model Assessment of Insulin Resistance; T, total testosterone

#### Cumulative meta-analysis

**Fig 4:**
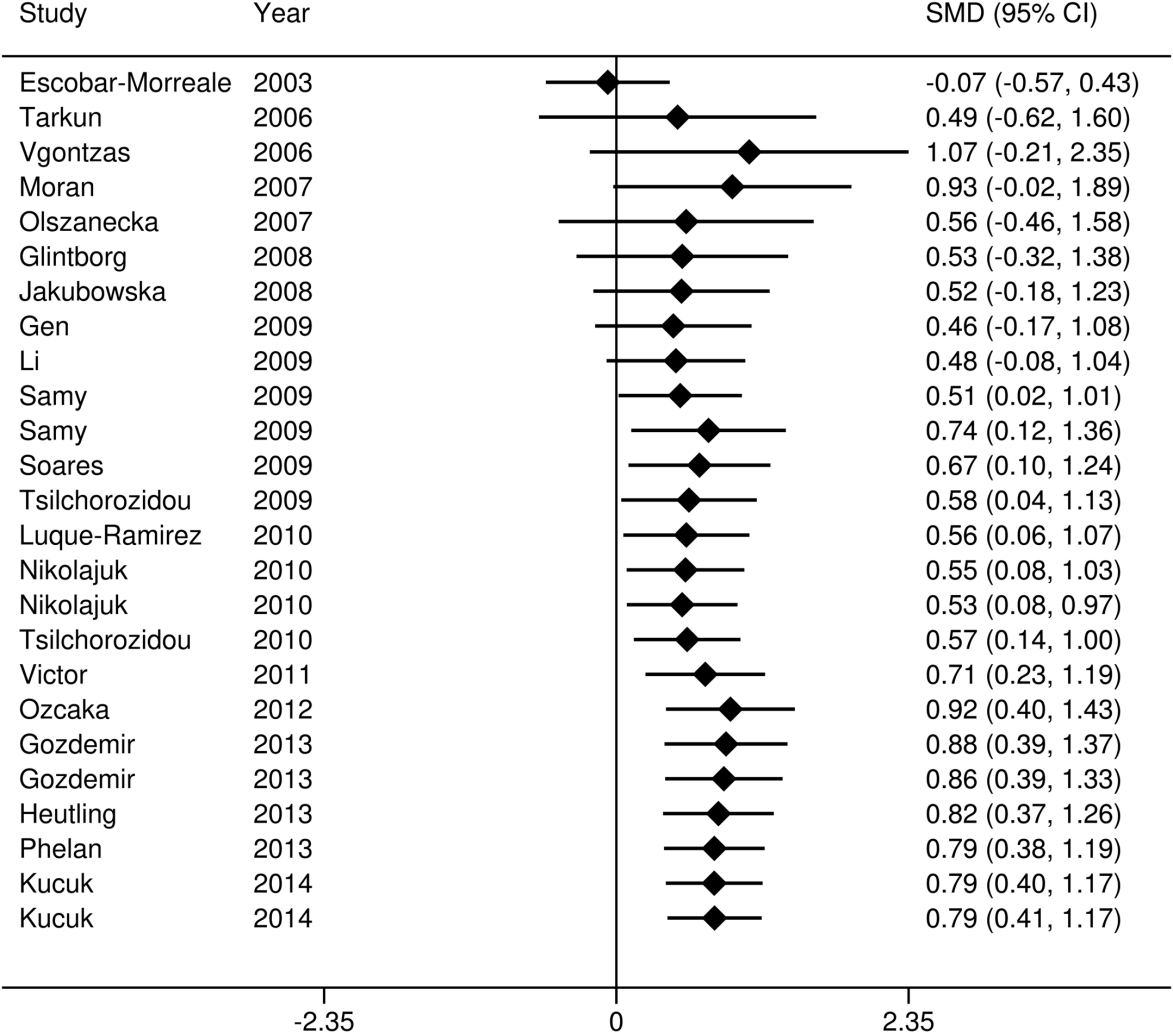
Cumulative meta-analysis.

To explore the evidence for changes in the IL-6 levels of women with PCOS over time, a cumulative meta-analysis was carried out (Fig 4). The results of the cumulative meta-analysis indicated that the levels of IL-6 were higher in those with PCOS compared with the controls. The total difference became statistically significance since added the study by Sammy et al. [15] (SMD = 0.51, 95% CI = 0.02–1.01), and the tendency of SMD remained stably since 2012 (0.79 to 0.92), which provided further evidence for differences in the IL-6 levels of the PCOS patients over time compared to the controls.

#### Sensitivity analysis

A sensitivity analysis was performed by omitting each study, one at a time, and observing the change in the pooled SMDs and 95% CIs. The study by Ozcaka *et al*. [18]was omitted first, which showed an impressive positive result, and the corresponding SMD and 95% CI were not significantly changed (SMD = 0.64, 95% CI = 0.29–0.98, *P*<0.001). Second, the study [28]that reported significantly decreased IL-6 levels in women with PCOS compared with the controls was excluded, and the corresponding SMD and 95% CI were also not significantly changed (SMD = 0.84, 95% CI = 0.48–1.21, *P*<0.001).

Studies containing outliers that might cause heterogeneity [9, 15, 17, 18, 28, 29, 35] (S5 Fig), as confirmed by a Galbraith plot analysis, were then excluded. The IL-6 levels remained significantly higher in the PCOS patients compared to the controls (SMD = 0.37, 95% CI = 0.22–0.53, *P*<0.001), but the heterogeneity decreased significantly (*I*^*2*^ = 23% and *P* = 0.200).

## Discussion

In this systematic review and meta-analysis of relevant studies, the levels of IL-6 were significantly higher in women with PCOS compared to BMI-matched controls. Higher IL-6 levels are related with the IR and total testosterone levels observed in women with PCOS compared with controls. Interestingly, the levels of IL-6 were high in both lean and obese women with PCOS. Significant heterogeneity was observed across the studies, and the level of IR was the main source of this heterogeneity.

### Comparison with Previous Meta-Analyses

The results of the present study are in contrast with those of previous studies [30, 31]. One of the included studies [30], which contained findings on 10 studies and 852 PCOS patients, found no significant differences in the serum IL-6 levels of PCOS patients compared with controls (15% difference, 95% CI = −15–45%, P = 0.331)[30]. Another study of the potential role of IL-6 levels as a marker of cardiovascular disease risk in women with PCOS also found no significant differences in the serum IL-6 levels of PCOS patients (WMD = 0.71, 95% CI = 0.16–1.59) [31]. That study included findings on 10 studies and 815 women with PCOS [31]. Differences between the present meta-analysis and the previous studies may contribute to the inconsistent results. First, the current meta-analysis included more eligible studies than previous studies (25 vs. 10), and the sample size (*N* = 1,618) was nearly twice that of previous studies. With the expansion of sample size, the corresponding statistical power can be increased. Second, the inclusion criteria of the current study were different from those of previous studies. Only studies that had strictly matched the BMI of cases and controls were included. The present study also used clearly defined criteria for the diagnosis of PCOS. Two of the previous meta-analyses [30, 31] included BMI-mismatched studies [36, 37]. Considering that the levels of proinflammatory cytokines are usually elevated in obesity [8], potential selection bias can influence the results in BMI- mismatched studies.

In addition, in the current meta-analysis, a subgroup analysis based on the main characteristics (BMI, IR, androgen status) of the PCOS patients was first performed. The meta-analysis revealed that the raised IL-6 levels in women with PCOS were related to the levels of IR and androgen. Furthermore, elevated IL-6 levels were found both in lean and in obese PCOS patients. Given the aforementioned factors, the present meta-analysis can be considered the most comprehensive and up-to-date study on serum IL-6 levels in women with PCOS.

### Implications for Clinical Practice

Although this study found no relationship between IL-6 levels and the BMI of PCOS patients, the data analysis pointed to a direct relationship between IR, androgen, and elevated IL-6 levels. The findings of the present study indicate that high IL-6 levels are not an intrinsic characteristic of PCOS. Therefore, IL-6 should not be used as a biomarker for the diagnosis of PCOS. However, the results found elevated IL-6 levels in the high HOMA2-IR ratio and T ratio subgroups, suggesting that the IL-6 levels increased with the severity of IR and the androgen status in PCOS patients. One study reported a significant decrease in IL-6 levels in women with PCOS [38], and two studies reported treatment-related reductions in IL-6 levels in patients with PCOS [26, 39]. Hence, the change in the serum IL-6 concentration may be a useful biomarker for the effect of treatment on PCOS, especially the effect of metformin on PCOS patients with T2DM. The insulin-sensitizing actions, anti-inflammatory actions, and antiatherogenic activity of IL-6 could be of potential research interest in PCOS.

### Limitations and Advantages

The main limitation of the present study is the significant heterogeneity across the included studies. This heterogeneity remained after the subgroup analysis, which could influence the conclusions of this meta-analysis. Moreover, a funnel plot of the data to explore publication bias was not produced because it was inappropriately applied for large heterogeneous across studies [40]. Therefore, potential publication bias should be considered. The technical measurements of IL6 should also be included as a factor for the heterogeneity analysis, however, it is harder to get the information of corresponding reagent from authors. In addition, SMD but not WMD was used to evaluate the differences in IL-6 levels. Thus, although the pooled results show statistical significance, they do not explain the clinical significance very well. Furthermore, missing data on the HOMA2-IR ratio and T ratio in some studies may produce a certain degree of systemic bias. Finally, most of the included studies had relatively small sample sizes. Larger studies are needed to strengthen the conclusions of the present study. These limitations must be considered when interpreting the results of this meta-analysis.

There are some advantages of this study. First, the statistical power of this meta-analysis is relatively better than that of previous studies. In addition, it is the most comprehensive study to date, as it investigated the impact of BMI, IR and androgen status of PCOS on IL-6 levels. Second, various methods were used to explore the source of the heterogeneity. The exclusion of the studies responsible for the heterogeneity had only an extremely small effect on the results. Furthermore, the pooled results were similar after excluding the outliers confirmed by a Galbraith plot analysis, and the heterogeneity was significantly decreased (*I*^*2*^<25% and *P*>0.1). In addition, the results of the cumulative meta-analysis indicated that the 95% CIs decreased in accordance with an increase in the number of studies included. All the aforementioned factors suggest that the results of this meta-analysis are statistically stable.

## Conclusions

This meta-analysis suggested that IL-6 levels were higher in women with PCOS compared with BMI-matched controls and that a high serum IL-6 concentration was related to IR and androgen levels but not to the BMI. Therefore, a high IL-6 level is not an intrinsic characteristic of PCOS, although it may be a useful monitoring biomarker for the treatment of PCOS.

## Supporting Information

S1 Fig Forest plot for IL-6 levels in PCOS patients compared with controls stratified by HOMA-IR ratio. (TIF)

S2 Fig Forest plot for IL-6 levels in PCOS patients compared with controls stratified by testosterone ratio. (TIF)

S3 Fig Forest plot for IL-6 levels in PCOS patients compared with controls stratified by BMI. (TIF)

S4 Fig Forest plot for IL-6 levels in PCOS patients compared with controls stratified by study quality. (TIF)

S5 Fig Galbraith plots of IL-6 levels in PCOS patients compared with controls. (TIF)

S1 File The search strategy and excluded studies with reasons. (DOCX)

S1 Table PRISMA 2009 Checklist. (DOC)

S2 Table Scale for quality assessment. (DOCX)

S3 Table The data of studies included in the meta-analysis. (DOCX)
