# Supplementary figures and images for: Interleukin-6 Levels in Women with Polycystic Ovary Syndrome: A Systematic Review and Meta-Analysis
Source: PLoS One. 2016 Feb 5;11(2):e0148531. doi: 10.1371/journal.pone.0148531 (PMC4746122; doi:10.1371/journal.pone.0148531)

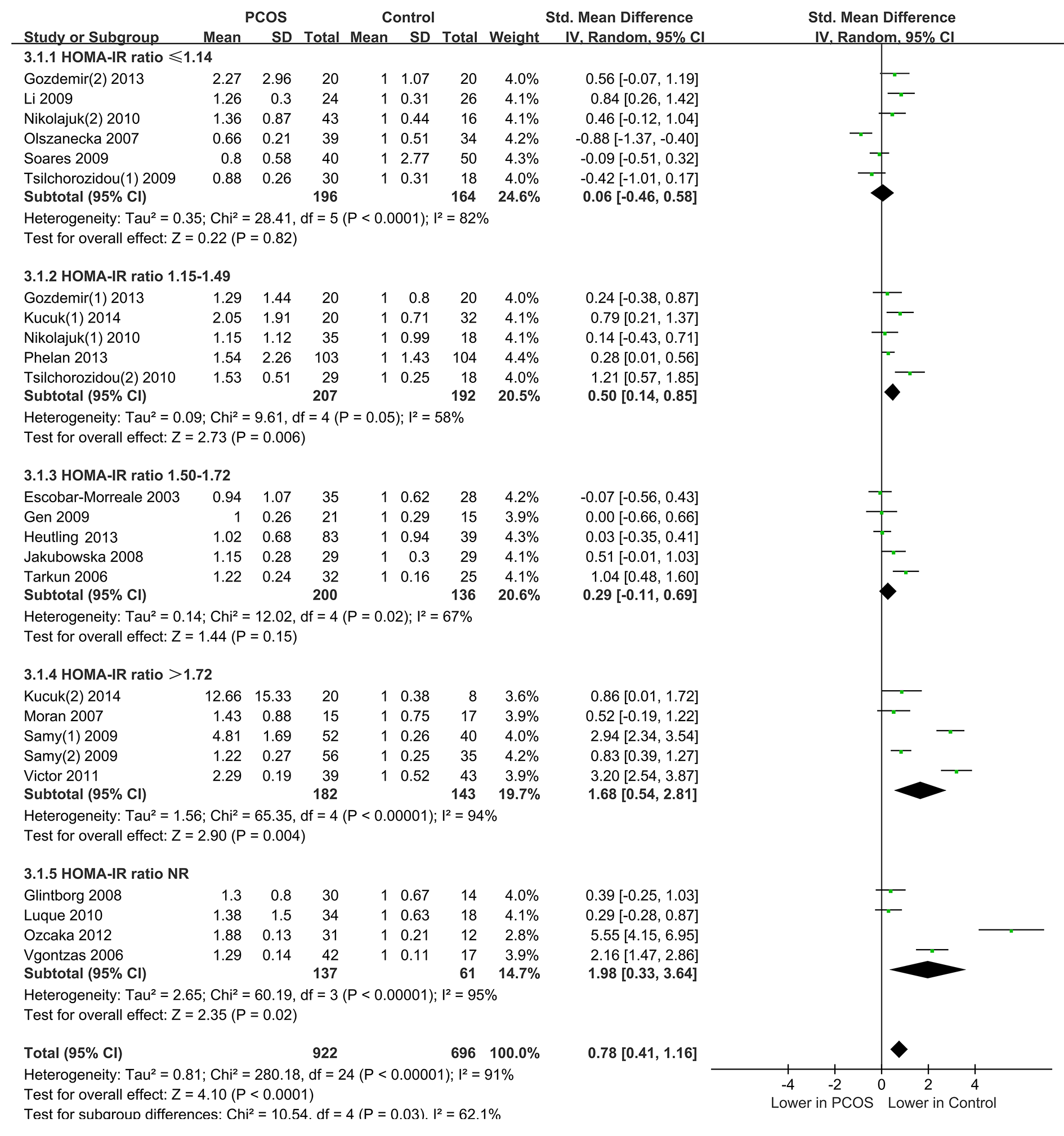

Supplement: S1 Fig — (TIF) [file pone.0148531.s001.tif]

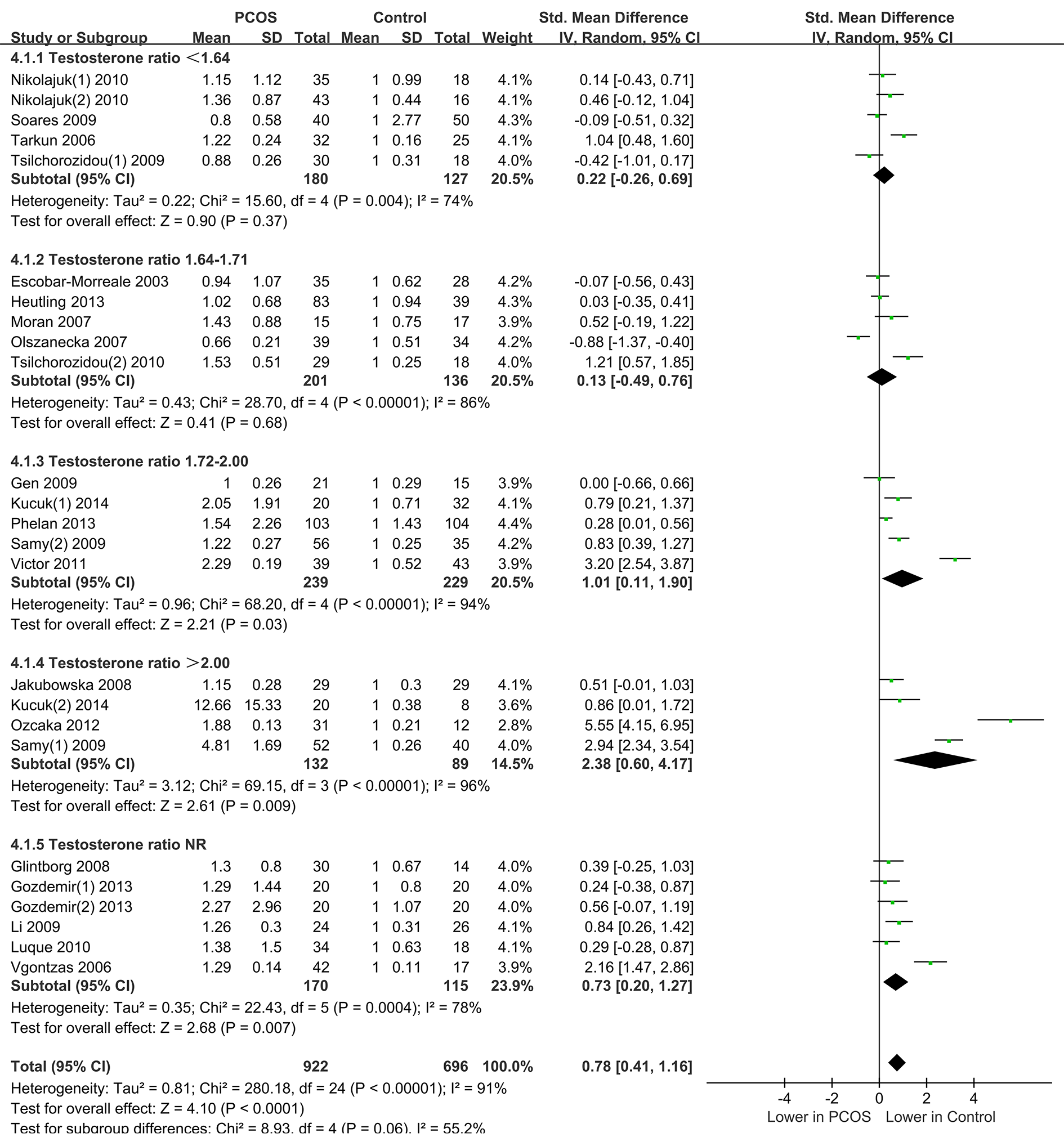

Supplement: S2 Fig — (TIF) [file pone.0148531.s002.tif]

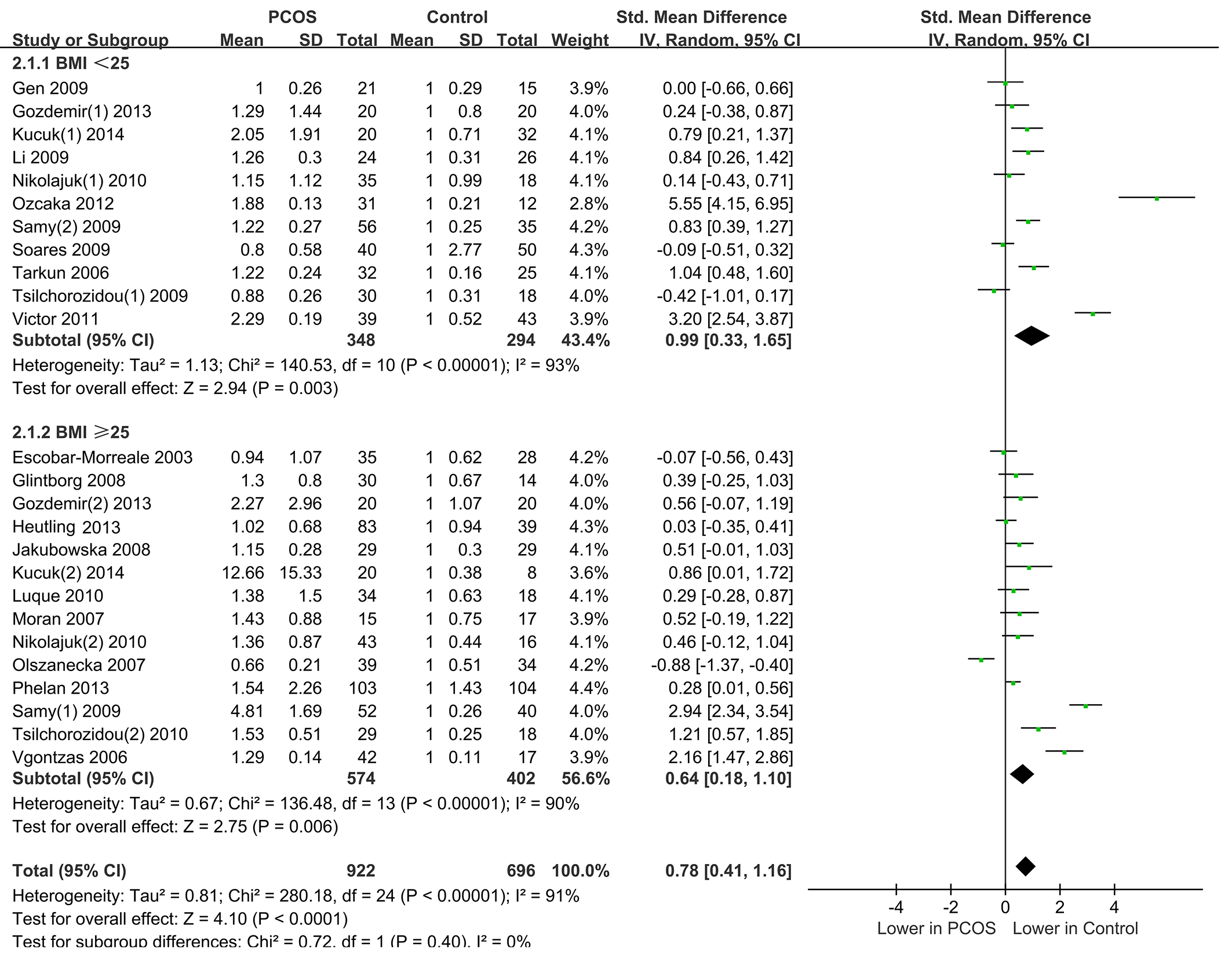

Supplement: S3 Fig — (TIF) [file pone.0148531.s003.tif]

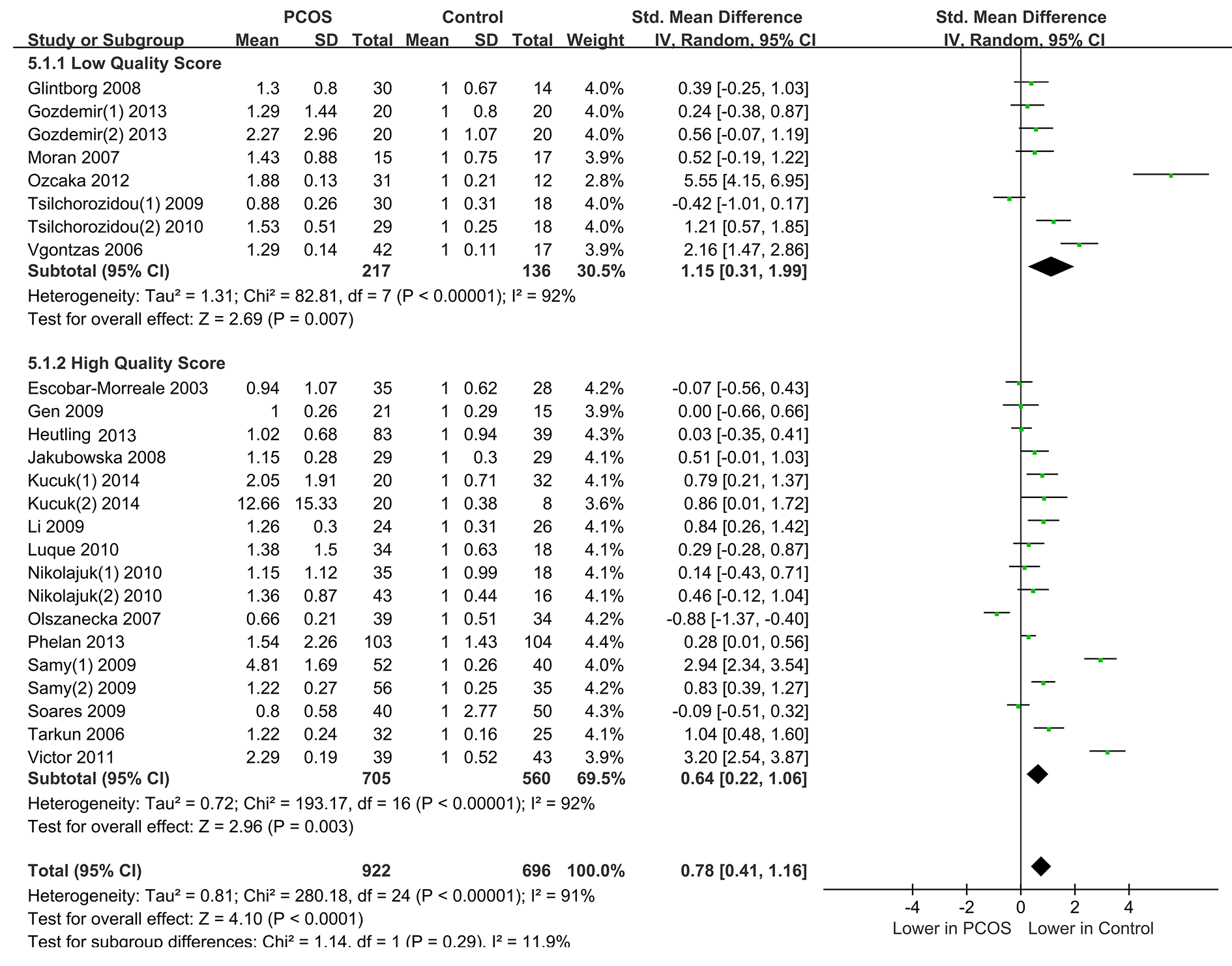

Supplement: S4 Fig — (TIF) [file pone.0148531.s004.tif]

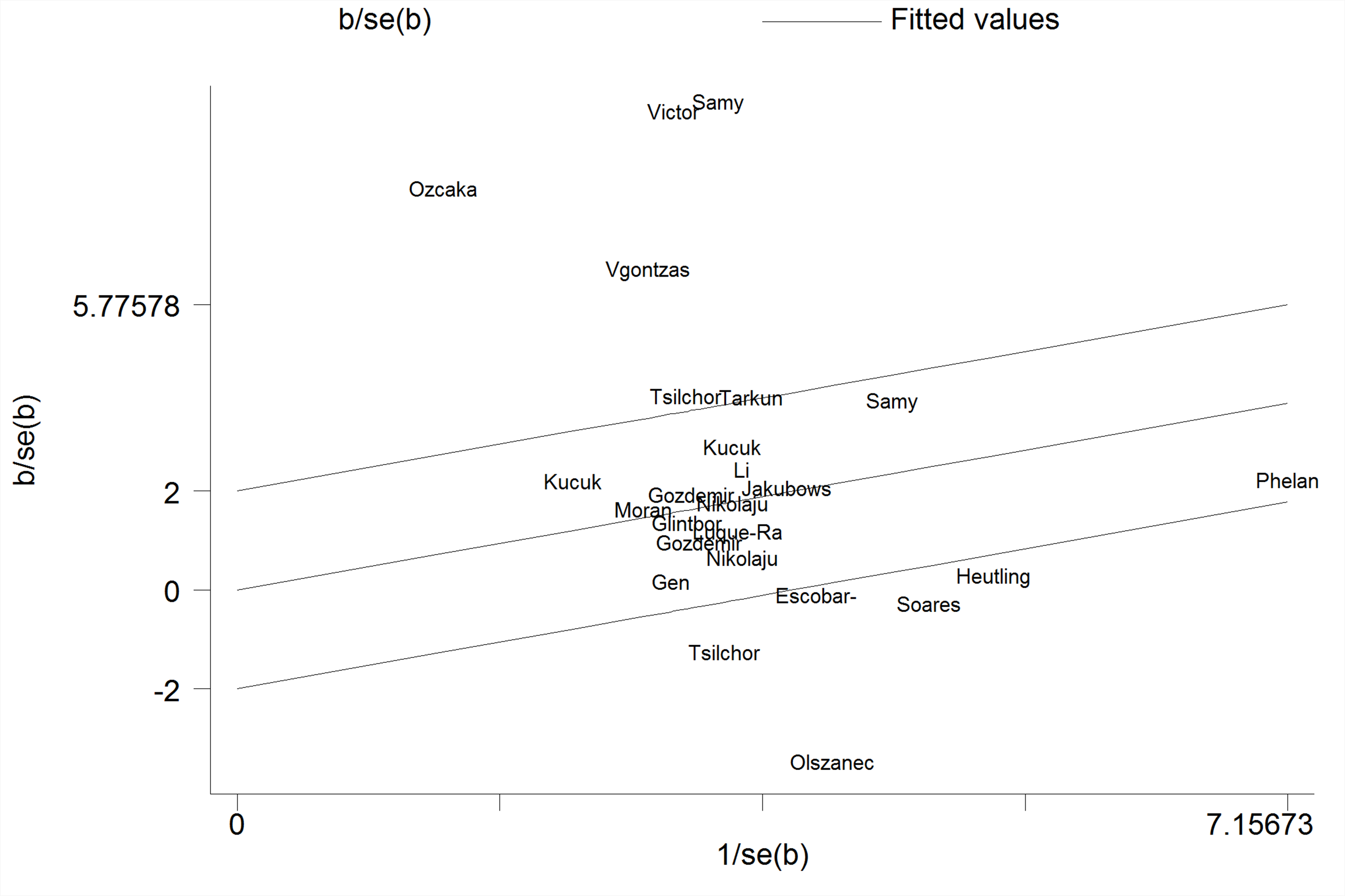

Supplement: S5 Fig — (TIF) [file pone.0148531.s005.tif]
