# Supplementary material for: Interleukin-6 Levels in Women with Polycystic Ovary Syndrome: A Systematic Review and Meta-Analysis
Source: PLoS One. 2016 Feb 5;11(2):e0148531. doi: 10.1371/journal.pone.0148531 (PMC4746122; doi:10.1371/journal.pone.0148531)
Supplement: S1 File — (DOCX) [file pone.0148531.s006.docx]

Search (((polycystic ovary syndrome[Title/Abstract]) OR PCOS[Title/Abstract])) AND (((IL 6[Title/Abstract]) OR Interleukin-6[Title/Abstract]) OR IL-6[Title/Abstract]) Filters: Humans

Excluded with reasons:

No data: [1-10]

No control：[11]

mRNA levels in PBMCs[12]

Sample<10[13]

BMI not matched：[14-17]

1. Ibanez L, Valls C, Marcos MV, Ong K, Dunger DB, De Zegher F. Insulin sensitization for girls with precocious pubarche and with risk for polycystic ovary syndrome: effects of prepubertal initiation and postpubertal discontinuation of metformin treatment. The Journal of clinical endocrinology and metabolism. 2004;89(9):4331-7. Epub 2004/09/10. doi: 10.1210/jc.2004-0463. PubMed PMID: 15356029.

2. Ibanez L, de Zegher F. Ethinylestradiol-drospirenone, flutamide-metformin, or both for adolescents and women with hyperinsulinemic hyperandrogenism: opposite effects on adipocytokines and body adiposity. The Journal of clinical endocrinology and metabolism. 2004;89(4):1592-7. Epub 2004/04/09. doi: 10.1210/jc.2003-031281. PubMed PMID: 15070917.

3. Chazenbalk G, Trivax BS, Yildiz BO, Bertolotto C, Mathur R, Heneidi S, et al. Regulation of adiponectin secretion by adipocytes in the polycystic ovary syndrome: role of tumor necrosis factor-{alpha}. The Journal of clinical endocrinology and metabolism. 2010;95(2):935-42. Epub 2010/01/22. doi: 10.1210/jc.2009-1158. PubMed PMID: 20089616; PubMed Central PMCID: PMCPmc2840865.

4. Fulghesu AM, Sanna F, Uda S, Magnini R, Portoghese E, Batetta B. IL-6 serum levels and production is related to an altered immune response in polycystic ovary syndrome girls with insulin resistance. Mediators of inflammation. 2011;2011:389317. Epub 2011/05/07. doi: 10.1155/2011/389317. PubMed PMID: 21547256; PubMed Central PMCID: PMCPmc3086286.

5. Svendsen PF, Christiansen M, Hedley PL, Nilas L, Pedersen SB, Madsbad S. Adipose expression of adipocytokines in women with polycystic ovary syndrome. Fertility and sterility. 2012;98(1):235-41. Epub 2012/05/23. doi: 10.1016/j.fertnstert.2012.03.056. PubMed PMID: 22607892.

6. Rey-Roldan E, Perez Lana MB, Galluzzo L, Blanco G, Onetto C, Straminsky V, et al. Is the polycystic ovary syndrome the causative of the increase in inflammatory markers and metabolic risk? Gynecological endocrinology : the official journal of the International Society of Gynecological Endocrinology. 2013;29(2):141-4. Epub 2012/11/03. doi: 10.3109/09513590.2012.730581. PubMed PMID: 23116196.

7. González F, Kirwan JP, Rote NS, Minium J. Evidence of mononuclear cell preactivation in the fasting state in polycystic ovary syndrome. American journal of obstetrics and gynecology. 2014;211(6):635. e1-. e7.

8. Knebel B, Janssen OE, Hahn S, Jacob S, Gleich J, Kotzka J, et al. Increased low grade inflammatory serum markers in patients with Polycystic ovary syndrome (PCOS) and their relationship to PPARgamma gene variants. Experimental and clinical endocrinology & diabetes : official journal, German Society of Endocrinology [and] German Diabetes Association. 2008;116(8):481-6. Epub 2008/08/06. doi: 10.1055/s-2008-1058085. PubMed PMID: 18680073.

9. Amato G, Conte M, Mazziotti G, Lalli E, Vitolo G, Tucker AT, et al. Serum and follicular fluid cytokines in polycystic ovary syndrome during stimulated cycles. Obstetrics and gynecology. 2003;101(6):1177-82. Epub 2003/06/12. PubMed PMID: 12798522.

10. Kucuk M, Altinkaya SO, Nergiz S, Sezer SD, Yuksel H, Bagli I, et al. Interleukin-6 levels in relation with hormonal and metabolic profile in patients with polycystic ovary syndrome. Gynecological endocrinology : the official journal of the International Society of Gynecological Endocrinology. 2014;30(6):423-7. Epub 2014/03/19. doi: 10.3109/09513590.2014.895981. PubMed PMID: 24628003.

11. Shroff R, Kerchner A, Maifeld M, Van Beek EJ, Jagasia D, Dokras A. Young obese women with polycystic ovary syndrome have evidence of early coronary atherosclerosis. The Journal of clinical endocrinology and metabolism. 2007;92(12):4609-14. Epub 2007/09/13. doi: 10.1210/jc.2007-1343. PubMed PMID: 17848406.

12. Seow KM, Juan CC, Wang PH, Ho LT, Hwang JL. Expression levels of vascular cell adhesion molecule-1 in young and nonobese women with polycystic ovary syndrome. Gynecologic and obstetric investigation. 2012;73(3):236-41. Epub 2012/03/24. doi: 10.1159/000334175. PubMed PMID: 22441212.

13. Gonzalez F, Rote NS, Minium J, Kirwan JP. Evidence of proatherogenic inflammation in polycystic ovary syndrome. Metabolism: clinical and experimental. 2009;58(7):954-62. Epub 2009/04/21. doi: 10.1016/j.metabol.2009.02.022. PubMed PMID: 19375763; PubMed Central PMCID: PMCPmc2737595.

14. Lin YS, Tsai SJ, Lin MW, Yang CT, Huang MF, Wu MH. Interleukin-6 as an early chronic inflammatory marker in polycystic ovary syndrome with insulin receptor substrate-2 polymorphism. American journal of reproductive immunology (New York, NY : 1989). 2011;66(6):527-33. Epub 2011/08/02. doi: 10.1111/j.1600-0897.2011.01059.x. PubMed PMID: 21801267.

15. Xiong YL, Liang XY, Yang X, Li Y, Wei LN. Low-grade chronic inflammation in the peripheral blood and ovaries of women with polycystic ovarian syndrome. European journal of obstetrics, gynecology, and reproductive biology. 2011;159(1):148-50. Epub 2011/09/13. doi: 10.1016/j.ejogrb.2011.07.012. PubMed PMID: 21908093.

16. Benson S, Janssen OE, Hahn S, Tan S, Dietz T, Mann K, et al. Obesity, depression, and chronic low-grade inflammation in women with polycystic ovary syndrome. Brain, behavior, and immunity. 2008;22(2):177-84. Epub 2007/08/25. doi: 10.1016/j.bbi.2007.07.003. PubMed PMID: 17716857.

17. Mohlig M, Spranger J, Osterhoff M, Ristow M, Pfeiffer AF, Schill T, et al. The polycystic ovary syndrome per se is not associated with increased chronic inflammation. European journal of endocrinology / European Federation of Endocrine Societies. 2004;150(4):525-32. Epub 2004/04/15. PubMed PMID: 15080783.
