# Supplementary material for: Interleukin-6 Levels in Women with Polycystic Ovary Syndrome: A Systematic Review and Meta-Analysis
Source: PLoS One. 2016 Feb 5;11(2):e0148531. doi: 10.1371/journal.pone.0148531 (PMC4746122; doi:10.1371/journal.pone.0148531)
Supplement: S2 Table — (DOCX) [file pone.0148531.s008.docx]

| **S2 Table Scale for quality assessment** | |
| --- | --- |
| **Criteria** | **Score** |
| **1. Is the case definition adequate?** |  |
| Yes, with independent validation. | 1 |
| No, or no description. | 0 |
| **2. Are the cases representative of population?** |  |
| Yes. | 1 |
| No, potential for selection biases or no description. | 0 |
| **3. Selection of controls** |  |
| Draw from the same community as the reference group. | 1 |
| Draw from a different source or no description. | 0 |
| **4. Definition of controls** |  |
| Controls have no history of disease. | 1 |
| Controls have history of disease or no description. | 0 |
| **5. Are study controls adjusted for age and body mass index?** |  |
| Yes. | 1 |
| No, or no description. | 0 |
| **6. Are study controls adjusted for additional factor, such as drinking and smoking status?** |  |
| Yes. | 1 |
| No, or no description. | 0 |
| **7.Sample sizes** |  |
| <50 | 1 |
| >50 | 0 |
