# Supplementary material for: Interleukin-6 Levels in Women with Polycystic Ovary Syndrome: A Systematic Review and Meta-Analysis
Source: PLoS One. 2016 Feb 5;11(2):e0148531. doi: 10.1371/journal.pone.0148531 (PMC4746122; doi:10.1371/journal.pone.0148531)
Supplement: S3 Table — (DOCX) [file pone.0148531.s009.docx]

**Table S2. The data of included studies in meta-analysis**

|  |  | **BMI(kg/m^2^)** | |  | **Age(year)** | |  | **IL-6 Level(pg//ml)** | |  |  |
| --- | --- | --- | --- | --- | --- | --- | --- | --- | --- | --- | --- |
| **Study** | **Method** | **PCOS** | **Control** |  | **PCOS** | **Control** |  | **PCOS** | **Control** | **HOMA-IR ratio** | **T-ratio** |
| Escobar-Morreale | ELISA | 31.8±9.2 | 30.6±8.0 |  | 24.9±7.4 | 31.7±8.0 |  | 0.76±0.87 | 0.81±0.50 | 1.51 | 1.64 |
| Tarkun | ELISA | 23.46±3.06 | 22.9±2.97 |  | 23.71±3.9 | 24.1±3.7 |  | 3.83±0.75 | 3.13±0.51 | 1.53 | 1.54 |
| Vgontzas | ELISA | 38.7±1.4 | 36.9±1.0 |  | 29.6±0.9 | 35.7±1.0 |  | 4.72±0.5 | 3.65±0.4 | NR | NR |
| Moran | ELISA | 35.7±5.8 | 35.5±5.1 |  | 31.7±6.2 | 37.1±4.7 |  | 16.0±9.8 | 11.2±8.4 | 1.89 | 1.65 |
| Olszanecka | ELISA | 35.6±5.7 | 36.1±5.5 |  | 27.4±6.5 | 31.0±5.9 |  | 5.40±1.7 | 8.2±4.2 | 0.9 | 1.71 |
| Glintborg | ELISA | 33.1(25.2-43.9) | 33.2(23.8-46.4) |  | NR | NR |  | 1.84±1.13 | 1.42±0.95 | NR | NR |
| Jakubowska | ELISA | 35.32 ±5.07 | 32.94 ±6.13 |  | 28.24±6.27 | 31.55±7.36 |  | 34.32±8.28 | 29.92±9.04 | 1.65 | 2.87 |
| Gen | ELISA | 20.74±1.75 | 20.85±2.08 |  | 21.85 ± 4.06 | 23.46 ± 5.15 |  | 0.31±0.08 | 0.31±0.09 | 1.58 | 2 |
| Li | ELISA | 20.3±1.9 | 20.6±2.1 |  | 25±5 | 27±7 |  | 0.184±0.044 | 0.146±0.045 | 1 | NR |
| Samy | ELISA | 32.2±1.1 | 31.4±1.2 |  | 28.3±4.2 | 27.5±5.1 |  | 6.45±2.27 | 1.34±0.35 | 3.04 | 2.13 |
| Samy | ELISA | 22.7±0.6 | 22.5±0.5 |  | 28.7±5.2 | 26.9±5.4 |  | 1.52±0.34 | 1.25±0.31 | 2.29 | 1.81 |
| Soares | ELISA | 22.7±3.3 | 23.1±3.2 |  | 24.5±3.8 | 24.5±5.1 |  | 1.95±1.41 | 2.43±6.72 | 1.04 | 1.54 |
| Tsilchorozidou | ELISA | 22.5±2.1 | 22.4±1.6 |  | 27.8±5.0 | 29.2±5.1 |  | 0.96±0.28 | 1.09±0.34 | 0.98 | 1.32 |
| Luque-Ramirez | ELISA | 30±6 | 29±8 |  | 24±6 | 27±5 |  | 1.1±1.2 | 0.8±0.5 | NR | NR |
| Nikolajuk | ELISA | 21.71±1.81 | 22.19±1.92 |  | 24.11±3.94 | 26.33±5.56 |  | 0.85±0.83 | 0.74±0.73 | 1.39 | 1.63 |
| Nikolajuk | ELISA | 31.46±4.34 | 30.66±4.37 |  | 25.60±5.57 | 27.44±5.27 |  | 1.16±0.74 | 0.85±0.37 | 1.14 | 1.62 |
| Tsilchorozidou | ELISA | 31.3±4.4 | 31.3±5.6 |  | 31.7±6.1 | 33.7±4.4 |  | 2.36±0.79 | 1.54±0.39 | 1.49 | 1.67 |
| Victor | ELISA | 22.2±2.6 | 21.8±2.6 |  | 24.2±7.3 | 26.0±5.0 |  | 4.8±0.4 | 2.1±1.1 | 1.73 | 1.8 |
| Ozcaka | ELISA | 21.47±0.90 | 21.55±0.93 |  | 28.52±2.17 | 22.12±1.99 |  | 18.55±1.29 | 9.85±2.06 | NR | 2.54 |
| Heutling | ELISA | 30.4±5.9 | 29.1±4.8 |  | 27.8±4.7 | 27.8±5.6 |  | 1.65±1.10 | 1.62±1.52 | 1.72 | 1.67 |
| Gozdemir | ELISA | 21.74±2.29 | 21.5±1.41 |  | 24.52±3.94 | 25.31±3.26 |  | 2.20±2.45 | 1.70±1.36 | 1.39 | NR |
| Gozdemir | ELISA | 29.97±4.43 | 29.75±3.77 |  | 24.70±4.74 | 27.6±4.91 |  | 7.09±9.23 | 3.12±3.33 | 0.98 | NR |
| Phelan | ELISA | 33 (13) | 31 (7) |  | 28 (8) | 34 (10) |  | 1.11±1.63 | 0.72±1.03 | 1.42 | 1.77 |
| Kucuk | ELISA | NR | NR |  | 23.8±5.6 | 24.9±5.2 |  | 28.25±26.26 | 13.75±9.72 | 1.29 | 2 |
| Kucuk | ELISA | NR | NR |  | 23.3±5.4 | 29.3±7.1 |  | 167.75±203.15 | 13.25±5.07 | 2.13 | 2.54 |
